# Variation of winter wheat phenology dataset in Huang Huai Hai Plain of China from 1981 to 2021

**DOI:** 10.1038/s41597-025-05368-z

**Published:** 2025-07-12

**Authors:** Quan-Jun Zhang, Dong-Li Wu, Jing Gao

**Affiliations:** 1 Meteorological Observation Centre, China Meteorological Administration (CMA), Beijing, 100081 China; 2 National Meteorological Information Center, Beijing, 100081 China

**Keywords:** Phenology, Plant ecology

## Abstract

This study presents a comprehensive analysis of winter wheat phenological variations in China’s Huang-Huai-Hai Plain (HHHP) from 1981 to 2021, leveraging data from 62 national agrometeorological observation stations. As the world’s largest winter wheat production region, the HHHP contributes over 60% of China’s total output, playing a pivotal role in national food security. Using kernel density estimation (KDE) and univariate linear regression, the dataset characterizes interannual trends in key phenological stages—sowing, emergence, tillering, jointing, booting, heading, flowering, milking, and maturity—along with growth period durations. Results reveal significant shifts in phenological timings and growth stages under climate change, such as advanced heading stages and altered phase lengths, which correlate with temperature increases and extreme weather events. The dataset, comprising 1,120 figures generated via Origin Lab, is publicly available on ScienceDB, providing critical insights for climate adaptation strategies, cultivation optimization, and yield stability. Technical validation confirms the reliability of the data, sourced from standardized, long-term manual observations by trained professionals under China Meteorological Administration protocols. This work offers a foundational resource for understanding climate-crop interactions and guiding sustainable agricultural practices in a warming world.

## Background & Summary

Winter wheat plays an indispensable role in global food security, and its stable and high-yield characteristics make it a crucial source of grain reserves for many countries^1,2^. Approximately 75% of the world’s wheat production comes from winter wheat, a proportion that underscores its central role in the global food supply^3,4^. Against the backdrop of a continuously growing population, the stable supply of winter wheat is crucial to meeting global food demand. Winter wheat, with its growth cycle spanning four seasons, accumulates more nutrients such as proteins and lipids, making it a vital source of energy and nutrition. Its grains are rich in carbohydrates, cellulose, and minerals, supporting the staple food needs of 35%–40% of the global population^2,5,6^.

As the world’s largest producer of winter wheat, China cultivates this crop mainly south of the Great Wall, spanning regions including North China and the Huang-Huai-Hai area. Winter wheat production represents over 56% of China’s total wheat output^6^. In 2023, China’s wheat cultivation area reached approximately 23.6272 million hectares, with winter wheat accounting for the vast majority. Such large-scale cultivation has provided stable income sources for numerous farming households and driven the development of related agricultural industries, including seeds, fertilizers, pesticides, and agricultural machinery. This has significantly contributed to the prosperity of the rural economy^7,8^.

Climate change has had a significant impact on the growth and yield of winter wheat. Global warming has led to changes in the growing conditions of winter wheat, thereby affecting its yield and quality^9,10^. Rising temperatures have shortened the growth cycle of winter wheat, potentially resulting in insufficient accumulated thermal units during critical growth stages, thereby impairing normal development^11,12^. For instance, in some regions, the accelerated jointing and booting stages of winter wheat expose the crop to heat stress during late growth phases, negatively impacting grain filling and kernel plumpness^13,14^. In recent years, extreme weather events such as droughts, floods, heatwaves, and cold spells have become increasingly frequent, posing serious threats to the growth and yield of winter wheat^15,16^. For example, the late July 2023 rainstorm in Beijing triggered severe waterlogging in some fields, severely compromising crop growth of winter wheat^17^.

Accurate monitoring and regulation of winter wheat phenological stages are critical for ensuring high and stable yields, optimizing resource utilization, and adapting to climate change, representing a core research focus^18,19^. Phenological stages serve as the “biological clock” governing winter wheat’s responses to environmental variations^18,20^. Under the background of global warming, the phenological stages of winter wheat have generally advanced. For example, in North China over the past 30 years, the heading stage of winter wheat has occurred 5–8 days earlier on average, making it more vulnerable to late frost damage and high-temperature stress during the grain-filling period^21,22^. Investigating the dynamic interactions between phenological stages and temperature, precipitation, and photoperiod can provide a scientific basis for crop breeding and optimization of cultivation zoning, thus ensuring spatiotemporal matching between crops and climate resources. The physiological processes at different phenological stages directly govern yield components. For example, the number of effective tillers during the tillering stage influences panicle count, photosynthetic assimilation efficiency from jointing to heading stages determines grains per panicle, while the duration and intensity of the grain-filling stage correlate with thousand-grain weight^23,24^.

Moreover, critical phenological stages exhibit significant temporal correlations with meteorological disaster windows^25,26^. For instance, exposure to −2 °C temperatures post-jointing stage can induce panicle development impairment^27^, while prolonged rainy spells during flowering stage may trigger Fusarium head blight^28^. Dynamic monitoring of phenological stages, when integrated with meteorological forecasts, enables the establishment of region- and stage-specific disaster early-warning systems. The phenological shifts in winter wheat have been extensively utilized in assessing long-term impacts of climate change on agricultural systems^29^. European studies indicate that each 1-day advancement in heading stage due to warming increases yield fluctuation risks by 3%–5%^30^, while phenological shifts in winter wheat across the North American Great Plains have led to mismatches with pollinator activity periods^31^. Such research provides empirical support for cross-regional adaptive policy formulation, including sowing date adjustments and insurance mechanism design. Winter wheat phenology research serves as a pivotal hub connecting genetic potential, environmental responses, and agronomic practices, with its outcomes not only advancing crop science theory but also directly contributing to food security and sustainable agriculture.

The Huang-Huai-Hai Plain, China’s largest concentrated winter wheat production region, contributes over 60% of the nation’s total winter wheat output, serving as the core “breadbasket” for national food security^32–34^. This study employs kernel density estimation and linear trend analysis on winter wheat phenological data from 62 national ago-meteorological observation stations across the Huang-Huai-Hai Plain during 1981–2021. Using Origin Lab, 1120 illustrative figures were generated to characterize the interannual variations in winter wheat developmental stages and their durations, aiming to provide theoretical foundations and scientific support for climate change adaptation research and cultivation management in winter wheat production.

## Methods

### Agrometeorological observation stations and data

**Fig. 1:**
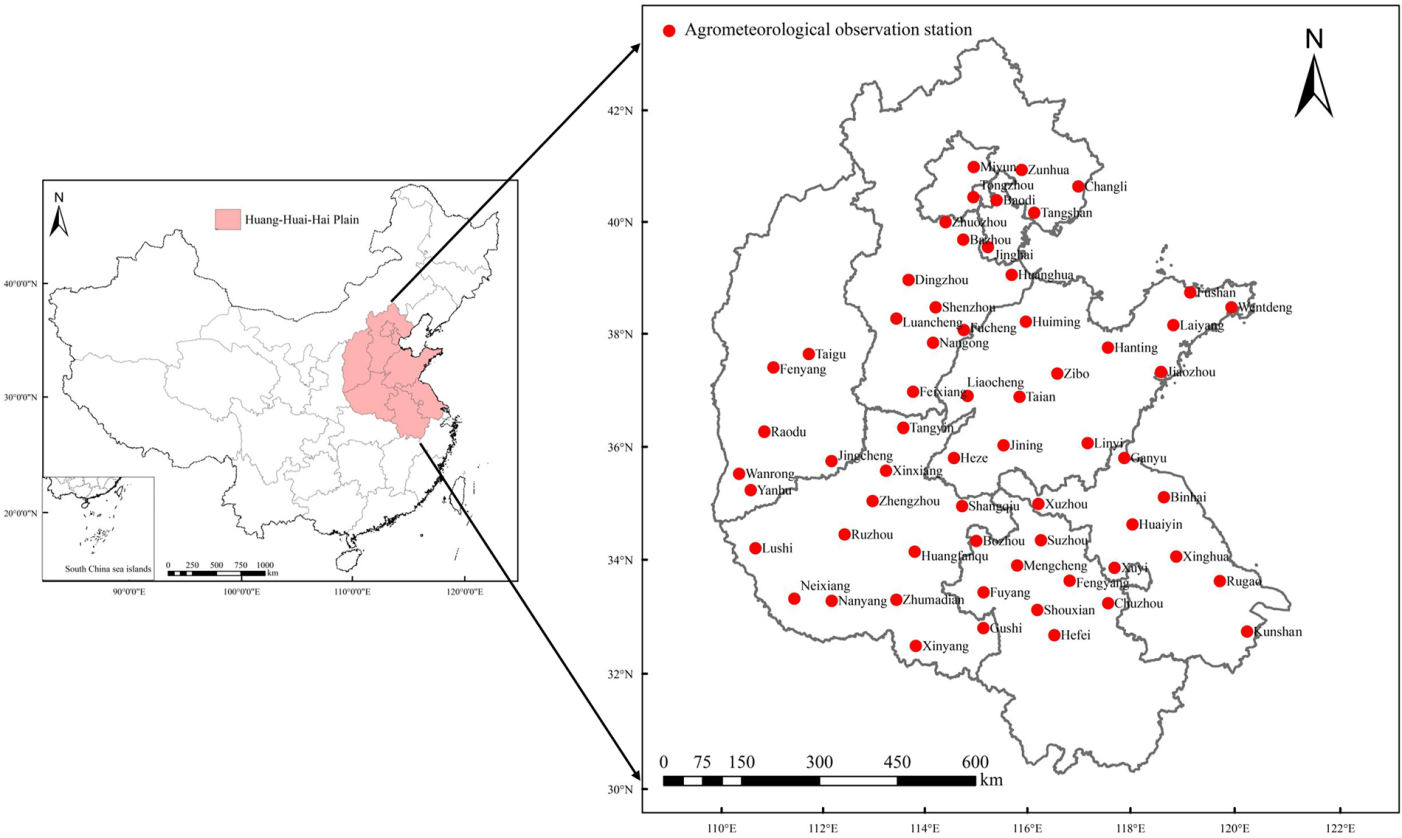
Spatial distribution of 62 agrometeorological observation stations included in the dataset.

Our dataset includes the records collected from 1981 to 2021 of 62 agrometeorological observation stations in the HHHP, including Hebei, Henan, Shandong, Shanxi, Jiangsu and Anhui provinces, and Beijing and Tianjin Municipalities. These stations were maintained by the Chinese Meteorological Administration to investigate local crop production and agricultural meteorological disasters, as well as provide suggestions for local farmers to manage agricultural meteorological disasters. There were 2 stations in Beijing Municipalities, 2 stations in Tianjin Municipalities, 12 stations in Hebei province, 6 stations in Shanxi province, 8 stations in Jiangsu province, 8 stations in Anhui province, 12 stations in Shandong province, 12 stations in Shandong province, and 12 stations in Henan province (Fig. 1). All stations are located in a winter wheat primary production region, and represent the typical cropping system in the HHHP. They are geographically and climatologically different, and have good records of crop data during1981–2021.These stations provide a unique chance to investigate the long series phenology variation trend characteristics.

**Fig. 2:**
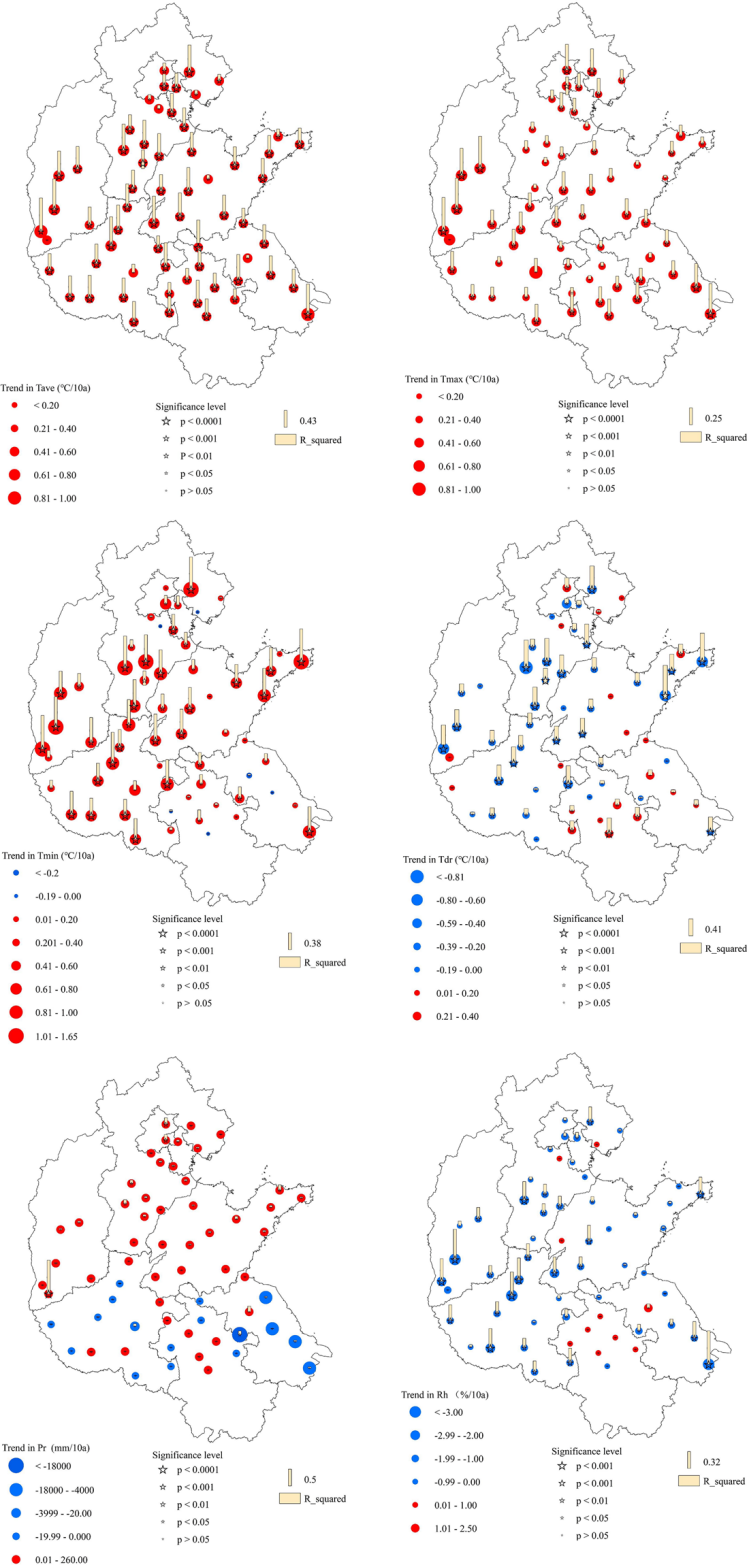
Characteristics of agrometeorological parameters of 62 agrometeorological observation stations.

The agrometeorological parameters, including average temperature (Tave), maximum temperature (Tmax), minimum temperature (Tmin), daily temperature range (Tdr), relative humidity (Rh) and precipitation (Pr), of these agrometeorological observation stations in recent 40 years were obtained from the official channel of the China Meteorological Administration, analyzing the historical change characteristics of these meteorological parameters (Fig. 2). In the past 40 years, the agrometeorological parameters of the observation stations in the HHHP had changed significantly, the Tave, Tmax, Tmin, and Pr had shown a significant upward trend, while the Dtr and Rh had shown a downward trend.

### Dataset construction process

**Fig. 3:**
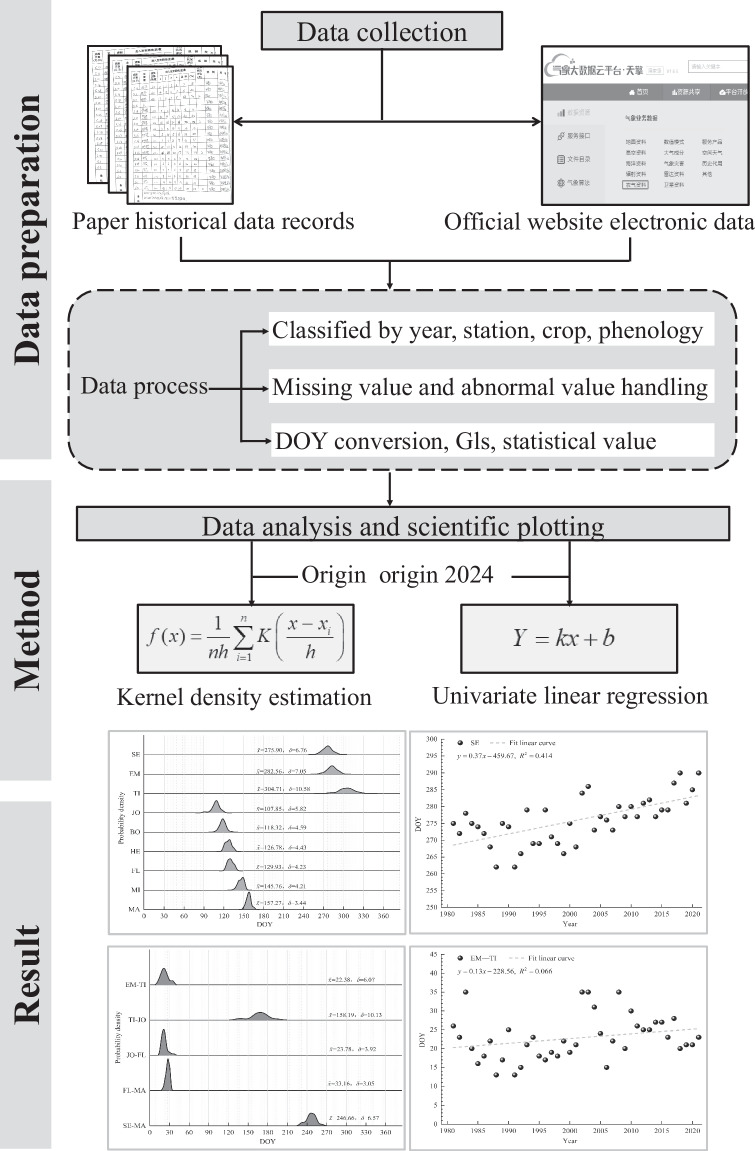
Flowchart construction process the dataset winter wheat phenology dataset in HHHP.

The dataset construction process includes the following key steps, as introduced in Fig. 3:

1. Data collection: Historical data and electronic data were collected from official archives and official website of Chinese Meteorological Administration. Winter wheat observed data from 1981 to 2021 for these stations included phenology, yields, cultivar and agronomic management practices. The detailed phenological records, including the dates of wheat sowing(SE), emergence(EM), tillering(TI), jointing(JO), booting(BO), heading(HE), flower (FL), milking(MI) and maturity(MA).
2. Data process: Firstly, the collected data are categorized by year, site, crop, and phenology. Then, quality control methods—such as historical paper-based data review, expert judgment based on professional experience, estimation using adjacent years’ data, and threshold checks—are applied to fill in missing values and correct anomalies. Finally, the data undergo processing steps including day of year (DOY) conversion, calculation of growth period length (GLs), and statistical analysis (e.g., mean and standard deviation computation).
  We took January 1 of each year as 1, and converted the date of the universal period of each phenology period to the day of year (DOY). According to the growth and development characteristics of each stage, the whole growth period length (GLs) was divided into 5 stages, namely, emergence to tiller (EM-TI), tiller to jointing (TI-JO), jointing to flowering (JO-FL), flowering to maturity (FL-MA) and whole growth period (SE-MA, WGP).
3. Data analysis and scientific plotting: The data processing, the calculation and analysis of the DOY were all completed in Excel 2023, and the kernel density estimation, univariate linear regression estimation and its significance test and plotting are all completed in Origin Lab origin 2024.

### Kernel density estimation

**Fig. 4:**
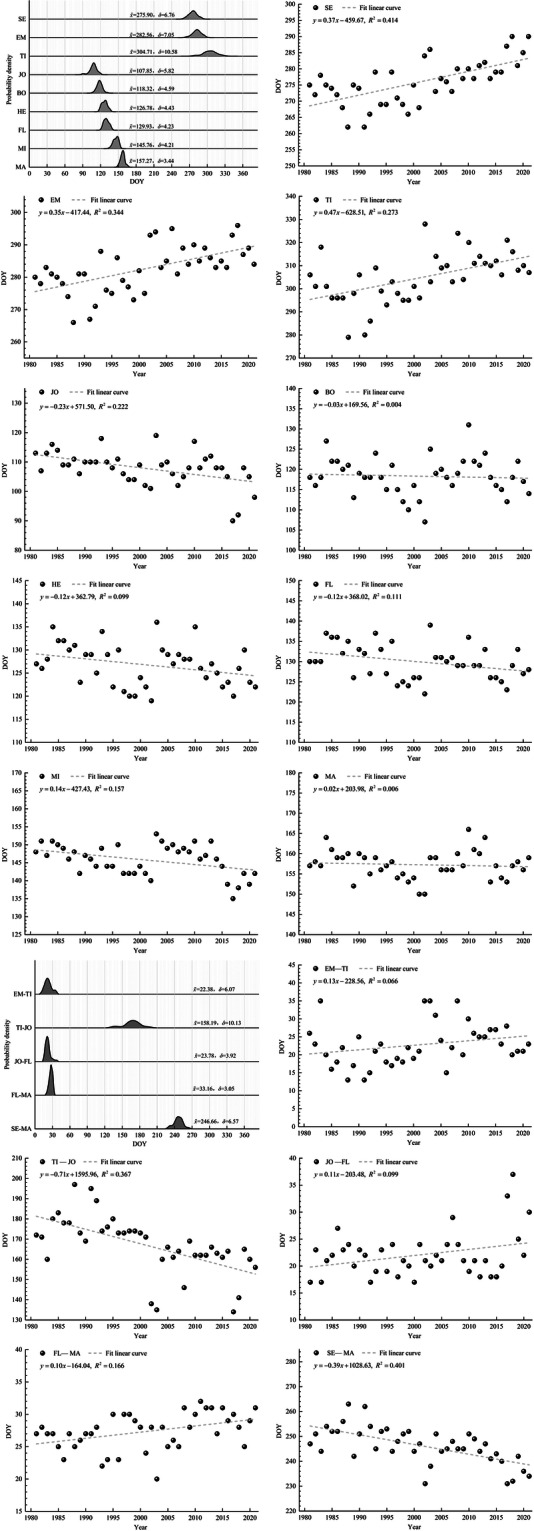
Example dataset: Forty-year (1981–2021) variation characteristics of winter wheat phenology and its lengths at Huanghua Agrometeorological Observation Station, Hebei Province.

We used the kernel density estimation (KDE) method to estimate the winter wheat phenological records of these stations. The average value and standard deviation of 40 years were statistically analyzed. We used the results of kernel density estimation and statistical to produce scientific illustrations as the core of this dataset. KDE is one of the nonparametric test methods used to estimate the unknown density function in probability theory. It can estimate the population from samples. KDE does not need to use the prior knowledge of data distribution, does not attach any assumptions to the data distribution, and only studies the characteristics of data distribution from the data sample itself, which has been highly valued in the field of statistical theory and application. Compared with other density estimation methods such as histogram, the probability density obtained by KDE has better continuity and does not depend on the selected interval length. The basic expression of KDE function is Fig. 4:

1 $$f(x)=\frac{1}{nh}\mathop{\sum }\limits_{i=1}^{n}K\left(\frac{x-{x}_{i}}{h}\right)$$

2 $$h=1.059\cdot \delta \cdot \frac{1}{\sqrt[5]{n}}$$

Where, *x*_1_, *x*_2_, *x*_3_,…, *x*_*n*_ represents the total number of DOY samples of *n* winter wheat at a certain phenology; K is the kernel function; H is bandwidth (automatically selected via Scott’s rule by Origin Lab 2024); *δ* is standard deviation; *f(x)* represents the probability density value of the DOY *x* estimated by KDE. The greater the value, the greater the probability that the DOY *x* is the DOY of a certain phenology.

### Univariate linear regression

The univariate linear regression method was used to analyze the variation trend of the DOY and its lengths. The univariate linear regression was estimated by the least square method. The model is as follows:

3 $$Y=kx+b$$

Where, *Y* represents the DOY or the GLs, and *x* is the year; *b* represents the intercept; represents the linear trend term, and *k* × 10 represents the 10-year trend of DOY or GLs. Negative *k* indicates that the phenology date is advanced or the length of GLs is shortened, and positive *k* indicates that the phenology date is delayed or the GLs is extended. Two tailed t-test analysis was used to determine the statistical significance of the change trend. The significance level for all regression analyses was set at α = 0.05. The regression analysis revealed p-values < 0.01 for key variables.

## Data Records

The Variation of winter wheat phenology dataset in Huang Huai Hai Plain of China from 1981 to 2021 is available at ScienceDB^35^. The dataset is provided in JPG format estimated and plotted by Origin Lab. All the diagrams can be downloaded directly for free. The dataset profile is shown in Table 1.

**Table 1:** Dataset profile.

Title	Variation of winter wheat phenology dataset in Huang Huai Hai Plain of China from 1981 to 2021
**Data corresponding author**	ZHANG Quan-Jun
**Data author(s)**	ZHANG Quan-Jun, WU Dong-Li, Gao Jing
**Time range**	1981–2021
**Geographical scope**	The administrative scope of the Huang-Huai-Hai Plain roughly encompasses the entirety of Beijing, Tianjin, and Shandong, the majority of Hebei and Henan provinces, as well as the northern Huai River regions of Jiangsu and Anhui provinces. The geographic latitude and longitude ranges are about 33°30′–45°40′, 119°10′–112°20′ respectively.
**Data analysis method**	Kernel density estimation and Univariate linear regression
**Data volume**	1.52 GB, 1120 diagrams
**Data format**	*.jpg
**Data service system**	10.57760/sciencedb.23011
**Source(s) of funding**	National Key R&D Program of China(2024YFD2301301); Innovative Development Special Project of China Meteorological Administration (CXFZ2023J069); Agricultural Observation Technology Innovation Team Project of Meteorological Observation Centre of China Meteorological Administration
**Dataset composition**	This dataset includes the kernel density estimation diagram and linear trend estimation diagram of winter wheat observation data at 9 phenology and 5 phenology stages from 62 national agrometeorological observation stations in Huang-Huai-Hai Plain in recent 40 years, which are put in 8 sub files, the total folder is named “HHHP- winter wheat phenology data”, with a total of 1120 diagrams.
**Dataset structure**	The first level folder is named “HHHP- winter wheat meteorology data”, which includes secondary folders named after 8 provinces or municipalities. Each secondary folders contains a tertiary folder named after the provincial average or observation station. Each tertiary folders contain 16 jpg diagrams, including 1 probability density diagrams of phenology, 9 linear fitting diagrams of DOY of phenology, 1 probability density diagrams of growth period length and 5 linear fitting diagrams of growth period length. The folder structures chart is as follow:

An example dataset is shown in Fig. 4. Figure 4 showed that the kernel density plots indicated the maximum probability density for SE, EM, TI, JO, BO, HE, FL, MI, and MA occurred on day 276, day 283, day 305, day 108, day 118, day 127, day 130, day 146, and day 157, respectively. The GLs EM–TI, TI–JO, JO–FL, FL–MA, and SE–MA exhibited maximum probability density at 22 days, 158 days, 24 days, 33 days, and 245 days, respectively. The linear regression results revealed the following trends: SE, EM and TI show significant delayed with rates of 3.7 days per decade (*R*^2^ = 0.414, *P* < 0.01), 3.5 d decade^−1^ (*R*^2^ = 0.344, *P* < 0.01), and 4.7 d decade^−1^ (*R*^2^ = 0.273, *P* < 0.01), respectively. JO, HE, and FL exhibit significant advancement with rates of −2.3 d decade^−1^ (*R*^2^ = 0.222, *P* < 0.01), −1.2 d decade^−1^ (*R*^2^ = 0.099, *P* < 0.01), and −1.2 d decade^−1^ (*R*^2^ = 0.111, *P* < 0.01), respectively. BO, MI, and MA showed no statistically significant changes. EM–TI and SE–MA shorten at rates of −7.1 d decade^−1^ (*R*^2^ = 0.367, *P* < 0.01) and −3.9 d decade^−1^ (*R*^2^ = 0.401, *P* < 0.01), respectively. JO–FL and FL–MA showed no significant trends.

## Dataset Values

This dataset analyzed the four-decade temporal variations in winter wheat phenology across 62 agrometeorological stations in diverse climatic zones of the HHHP.

In scientific research, it revealed the spatiotemporal evolution patterns of winter wheat phenology under climate change, providing an empirical foundation for deciphering the driving mechanisms of temperature, precipitation, and other climatic factors on phenological shifts. It identifies latitudinal disparities and topographic influences on phenology, uncovered small-scale climate adaptation strategies, and supported global change ecology studies.

The integration of kernel density estimation and linear trend analysis in this dataset enabled the validation of crop model parameters, addressing the limitations of traditional mean-based analyses and improving model prediction accuracy, thereby enhancing simulations of winter wheat phenology under future climate scenarios.

In agricultural practice, this dataset guided agricultural timing by clarifying trends in key developmental stages (e.g., sowing, jointing, and flowering), helping farmers optimize planting schedules to mitigate meteorological disaster risks such as low-temperature rainfall or drought. By analyzing changes in GLs, it facilitated the selection of early-maturing or stress-resistant cultivars adapted to localized climate variability and informs regional winter wheat planting zonation. Furthermore, it aids in refining field management practices—for instance, adjusting fertilization strategies in response to shortened vegetative growth periods or intensifying pest monitoring during prolonged reproductive phases—to enhance yield and quality.

In summary, this high-precision, long-term dataset established a foundational framework for studying crop adaptability under climate change, integrating theoretical depth with practical applicability. Its multidimensional value not only enhances current agricultural productivity but also provided critical data support for addressing future climatic uncertainties in agricultural systems.

## Technical Validation

The work largely benefited from agrometeorological observation stations in major grain production areas in China constructed and operated the China Meteorological Administration. Agrometeorological observation business of China began in the 1950s. In 2009, 653 national agrometeorological observation stations were identified, and in 2023, 642 national agrometeorological observation stations were adjusted, distributed in important grain production areas of provinces or municipalities across the country.

Many years of national standardized manual observation have accumulated a large number of valuable manual observation data. More than 1200 agrometeorological observers of the China Meteorological Administration are professional trained and well-trained scientific and technological personnel. Most of them have been engaged in agrometeorological observation for more than 10 years. The observation and recording process is scientific and rigorous. The observers regularly carry out intermediate quality inspection, cyclic evaluation of data quality, detection and correction of errors, and the data quality is guaranteed. The observation data are collected in strict accordance with the national unified “Specification for Agrometeorological Observation”^36^. Basis for determining winter wheat phenology are shown in Table 2. The observation section is selected in the field with an area of more than 0.10 hm^2^. According to the shape of the field, it is divided into four equal small areas as four repeated observation points, with conspicuous signboards inserted. 25 plants (stems) are taken from each of the four small areas for each observation to be observed synchronously, once every two days, and patrol observation is carried out at the end of each month. According to the percentage of the number of plants (stems) entering the phenology in the total number of plants (stems) observed, each phenology is divided into the initial stage of phenology (greater than or equal to 10%) and the universal stage of phenology (greater than or equal to 50%). The phenology date used in this study is the date of reaching the universal phenology period.

**Table 2:** Basis for determining winter wheat phenology.

Phenology	Observation basis
Sowing(SE)	Actual sowing date.
Emergence(EM)	The first green leaf emerges from the coleoptile, measuring approximately 2.0 cm in length, and the rows become visibly distinct when viewed vertically (under the row-sowing/drilling method).
Tillering(TI)	The tip of the first tiller’s leaf emerges from the leaf sheath, measuring approximately 0.5–1.0 cm in length.
Jointing(JO)	The basal internode of the stem elongates and protrudes approximately 1.5–2.0 cm above the ground. At this stage, spike differentiation enters the floret differentiation phase.
Booting(BO)	The flag leaf has fully emerged from the leaf sheath.
Heading(HE)	The tip of the spike emerges from the flag leaf sheath, with some spikes curved and emerging laterally from the sheath (excluding the awns).
Flower (FL)	In the upper-middle spikelets of the ear, the florets open as their glumes separate, exposing the anthers and releasing pollen. During rainy or overcast weather, the outer glumes may fail to open naturally, requiring careful manual peeling of the glumes for observation.
Milking(MI)	The kernels in the upper part of the ear have reached their normal size and display a yellow-green coloration. Within the interior, white solid masses have formed, and the kernels are filled with a milky fluid.
Maturity(MA)	Over 80% of the kernels on the ear have turned yellow, with both the glumes and culms yellowing, while only the first and second internodes from the top retain a slightly green color.

Observational uncertainties in this dataset primarily may stem from:(1) Human judgment variability: subjective discrepancies in identifying crop growth stages (e.g., leaf emergence, tillering, or spike differentiation) across different observers. (2) Environmental influences: adverse weather conditions (e.g., rain or humidity) occasionally prevented natural opening of glumes during flowering, requiring manual intervention for accurate phenological assessment. (3) Instrumental limitations: Field measurements (e.g., internode length, kernel size) may exhibit minor errors due to manual tools (e.g., rulers) or sensor precision. (4) Historical data gaps: Missing records in archived paper-based datasets necessitated interpolation or expert validation.

To mitigate these uncertainties and enhance data reliability, we implemented a multi-layered quality control measure: (1) Training & Calibration: Observers were rigorously trained to follow Agrometeorological Observation to minimize subjective biases. Tools for measuring length were regularly calibrated. (2) Cross-Verification: Missing values were filled using adjacent years’ data or validated against historical records. (3) Expert Review: Ambiguous cases were resolved through consensus with agronomy experts. (4) Expert Checks: It mainly includes completeness checks, duplicate checks, cross-year consistency checks, station ID checks, temporal consistency checks, range checks, and internal consistency checks, among others.

## Data Availability

No custom code was created for the production of this dataset.
